# High density trans-admittance mammography development and preliminary phantom tests

**DOI:** 10.1186/1475-925X-11-75

**Published:** 2012-09-25

**Authors:** Mingkang Zhao, Hun Wi, Abu Hena Mostofa Kamal, Alistair Lee McEwan, Eung Je Woo, Tong In Oh

**Affiliations:** 1 Department of Biomedical Engineering, Kyung Hee University, Yongin-si, Gyeonggi-do, 446-701, Korea; 2 The School of Electrical and Information Engineering, The University of Sydney, NSW, 2006, Australia

**Keywords:** Trans-admittance, Conductivity spectrum, Breast cancer, Lesion detection, Admittance measurement, 84.37. + *q* Mammography in computer-aided diagnosis, 87.57*.rh* Electric impedance measurement, 84.37. + *q*

## Abstract

**Background:**

Malignant breast tumor tissue has a significantly different electrical impedance spectrum than surrounding normal tissues. This has led to the development of impedance imaging as a supplementary or alternative method to X-ray mammography for screening and assessment of breast cancers. However low spatial resolution and poor signal to noise ratio has limited the clinical application.

**Methods:**

In order to improve spatial resolution we developed a trans-admittance mammography (TAM) system including an array of 60×60 current sensing electrodes. We adopted a similar setup to X-ray mammography where the breast is situated between two holding plates. The top plate is a large solid metal electrode for applying a sinusoidal voltage over a range of frequencies from 50 Hz to 500 kHz. The bottom plate has 3600 current sensing electrodes that are kept at the ground potential. Currents are generated from the top voltage-applying electrode and spread throughout the breast, entering the TAM system through the array of current sensing electrodes on the bottom plate. The TAM system measures the exit currents through 6 switching modules connected to 600 electrodes each. Each switching module is connected to 12 ammeter channels which are switched sequentially to 50 of the 600 electrodes each measurement time. Each ammeter channel is comprised of a current-to-voltage converter, a gain amplifier, filters, an analog to digital converter, and a digital phase sensitive demodulator.

**Results:**

We found an average noise level of 38 nA, amplitude stability of less than 0.2*%*, crosstalk of better than -60 dB and 70 dB signal to noise ratio over all channels and operating frequencies. Images were obtained in time difference and frequency difference modes in a saline phantom.

**Conclusion:**

We describe the design, construction, and calibration of a high density TAM system in detail. Successful high resolution time and frequency difference images showed regions of interest with the expected admittivity changes in the frequency spectrum.

## Background

Experimental findings show that cancerous breast tissues have significantly higher admittivity values compared with those of normal breast tissues [1-4]. For the diagnosis of breast cancer, several bioimpedance techniques have been suggested as an alternative or supplementary method to X-ray mammography. The urgent need here is to assist in reducing the large number of biopsies resulting from mammography. To apply the bioimpedance technique for breast imaging, two different approaches of tomographic and projection imaging have been proposed.

Electrical impedance tomography (EIT) is a tomographic imaging method to visualize an admittivity distribution inside the human body [5,6]. EIT typically uses a ring of electrodes placed around the body of interest. Breast imaging using EIT was proposed by Larson-Wiseman (1998), Mueller *et al* (1999), Cherepenin *et al* (2001 and 2002), Kerner *et al* (2002) and Kao *et al* (2003) [7-12]. While previous systems sought to allocate rings of electrodes around the breast, the latest research efforts are focused on imaging the breast using a fixed planar array of electrodes (projection imaging) as imaging is highly sensitive to relative electrode positions. The idea of using a handheld probe with a planar array of electrodes was first suggested by Kao *et al* (2006) who used two radiolucent plates of planar electrode arrays with the breast situated between them [13,14]. This design allows a dual-mode operation of EIT and X-ray mammography in the X-ray mammography geometry. Based on a three-dimensional forward model of the mammography geometry, Choi *et al* (2007) proposed a linear conductivity image reconstruction algorithm with a regularization method [15]. For the same geometry, Boverman *et al* (2008) developed a linear admittivity image reconstruction algorithm for spectroscopic imaging in the range of 3 kHz and 1 MHz [16]. Kao *et al* (2008) reported promising results of their preliminary patient study by relating regional admittivity spectra registered to 3D mammograms [17]. More patient studies with improved hardware, software and algorithms are expected to validate the ability to differentiate benign and malignant lesions, and establish the clinical significance of impedance imaging in the diagnosis of breast cancer.

In 1978, Henderson and Webster suggested a frontal plane impedance camera, which is considered to be the first medical impedance imaging method. Applying a constant voltage on the frontal plane of the chest using a large electrode, current enters and spreads throughout the chest. By attaching an array of reference electrodes on the back and measuring the exit current through each one of them, we may draw a two-dimensional map of the current distribution, which can be interpreted as a projection image of the admittivity distribution. This frontal plane impedance camera initiated medical impedance imaging research, however it received much less attention than the tomographic impedance imaging method until a commercial system called T-Scan was introduced for adjunctive clinical uses with X-ray mammography [18,19]. In the T-Scan configuration, a patient holds a voltage electrode with one hand and a flat scan probe is placed on her breast. The probe includes an array of electrodes at zero volts. Measured exit currents from all electrodes of the scan probe provided a two-dimensional trans-admittance image, visualising the admittivity distribution inside the local breast region underneath the scan probe.

The diagnostic information from the T-Scan system lacks a sophisticated reconstruction method of lesion localization, hence Seo *et al* (2004) and Ammari *et al* (2004) developed a mathematical framework to analyze the trans-admittance map and suggested a non-iterative algorithm to extract core features of lesions from two maps with and without a lesion [20,21]. Noting that admittivity spectra of cancerous and normal breast tissues are different, Oh *et al* (2007) and Kim *et al* (2008) suggested a frequency-difference method and showed its feasibility from numerical simulations and phantom experiments [22,23]. For their experimental study, Oh *et al* (2007) developed a multi-frequency trans-admittance scanner (TAS) and a cylindrical scan probe with 320 current-sensing electrodes [22]. Limited by the radius of the scan probe, its field of view was restricted to 20*mm* and the spatial resolution of the trans-admittance maps was also limited by the relative small number of current-sensing electrodes.

In this paper, we describe a new design of the trans-admittance scanner for the mammography setup (TAM) with better spatial resolution. Instead of using the handheld voltage electrode and scan probe, we use two plates with the breast between them. The imaging setup is identical to X-ray mammography except that we may rotate the two plates for consecutive projection imaging at any angle. After describing its basic performance including the signal-to-noise ratio (SNR), stability, noise and crosstalk between measurement channels, we will show time-difference and frequency-difference TAM images of a breast phantom with a known admittivity distribution. We hypothesize that the regions of interest within the frequency-difference TAM images reflect the expected admittivity changes in the frequency spectrum.

## Methods

### System architecture

**Figure 1:**
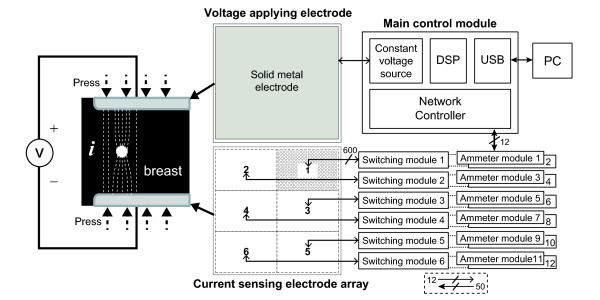
**Measurement configuration of the developed TAM system.** Measurement configuration of the developed TAM system. The breast is placed between two electrode plates both of which are pressed with controlled pressure against the breast in a similar setup to X-ray mammography systems. A controlled constant voltage is applied to the top electrode and exit currents measured through the bottom electrode array of 3600 electrodes. These are addressed in 6 banks of 600 electrodes. Each bank is switched to a pair of ammeter modules which consist of 6 independent ammeter channels each. The ammeter channels return the demodulated admittance to the main control module for reconstruction to an impedance image.

Figure 1 shows the measurement configuration and the structure of the TAM system. The breast is placed between two plates. We apply a constant sinusoidal voltage over a range of frequencies from 50 Hz to 500 kHz to the large top plate of 180×180*mm*stainless steel. The bottom plate consists of 3600 gold plated current sensing electrodes situated on a PCB. These are kept at the ground potential. From the potential difference between the two plates, current spreads throughout the breast and returns to the current sensing electrode array. Any conductivity anomaly distorts the current flow and its effect is mapped on the plane of current sensing electrodes. The measured exit current is varied by the trans-admittance distribution which is sensitive to the presence of lesions that create conductivity anomalies in the homogenous breast tissue. The exit current measurements can be reconstructed as a TAM image when considering the applied voltage. The two parallel electrode plates can be rotated around the breast to increase the measurement depth. The spatial resolution of measured trans-admittance maps is limited by the number and size of the current sensing electrodes. We chose the size of current sensing electrode array large enough to cover the whole breast when it is pressed. The TAM system comprises one voltage applying electrode, 3600 current sensing electrodes, 6 switching modules, 12 ammeter modules, a constant voltage source and a main control module including a network controller for communication between a PC and ammeter controllers as shown in Figure 1. Each functional block will be explained in following sections.

### Current sensing electrodes and switching module

**Figure 2:**
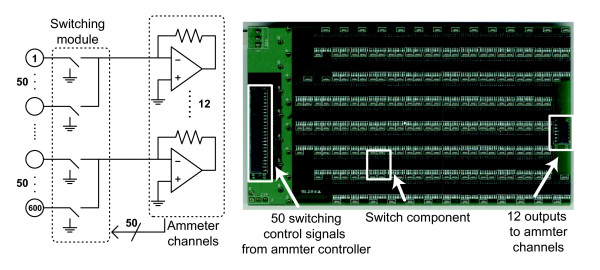
**The switching network.** The switching network between each 600 bank of current sensing electrodes and the pair of ammeter modules which contain a total of 12 independent ammeter channels.

The 3600 current sensing electrode array is connected to 6 switching modules each addressing 600 adjacent electrodes. Each current sensing electrode is a gold-coated, 2*mm* diameter, circular contact on a printed circuit board (PCB) with the commonly used Fire-Retardant glass laminate substrate (FR4). The spacing between adjacent electrodes is 3 *mm* center-to-center, leaving 1 *mm* gaps. On the back side of this PCB, we placed 18 surface mount connectors (MEG-array mezzanine connectors, FCI, France) for 3600 signal paths. Each connecter has 200 pins and it connects between each current sensing electrode and the corresponding input of a switch in one of the switching modules. At each measurement time, 12 signal outputs among the 600 electrodes are connected to 12 independent ammeter channels. This is repeated 50 times to cover all 600 electrodes. The 12 ammeter channels belong to one of the 2 ammeter modules shown in Figure 1 so that each ammeter module contains 6 ammeter channels. This arrangement was chosen to enable a compact and flexible design. Figure 2 shows the functional blocks for a switching module connected to 2 ammeter modules or 12 ammeter channels. The electrode output is switched either to the circuit ground or to the input of a chosen trans-resistance amplifier in the ammeter channel.

### Ammeter

**Figure 3:**
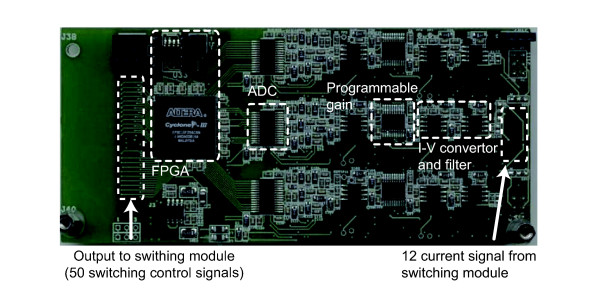
**Ammeter module.** Ammeter module with one ammeter channel highlighted. Each side of the PCB contains 3 ammeter channels. These consist of a trans-resistance amplifier (I-V converter), filter, programmable gain amplifier and ADC. Each ammeter channel sends the digitized current to the single FPGA for independent demodulation.

Two ammeter modules are responsible for 600 current sensing electrode outputs. One ammeter module consists of 6 ammeter channels, one of which is highlighted in Figure 3. The remaining 3 channels are located on the backside of this PCB. The exit current from a selected electrode is connected to the input of an ammeter channel. The front-end of each ammeter channel is a trans-resistance amplifier that converts the exit current from a selected electrode to the amplified voltage signal. The current to voltage (I-V) converter provides a virtual ground to the input for measuring the exit current [24]. We amplify the output of the I-V converter using a variable gain voltage amplifier and filter out the noise signal. A high speed analog to digital converter (ADC) quantizes the amplified and filtered signal for digital demodulation. The input dynamic range of the ammeter is ± 2.5 *mA* with minimum gain and the amplitude resolution is 3 nA with the maximum gain setting.

An ammeter controller for each ammeter module is implemented on a field programmable gate array (FPGA) (EP3C10F256C8N, Altera, USA) including 6 independently operating digital phase sensitive demodulators to calculate real and imaginary components of measured signals in parallel, and other control logic. The demodulator outputs are transferred to a PC for presenting images.

One ammeter controller is the master which is responsible for the generation of the 50 switching control signals that change the switch configuration in synchrony with the data acquisition time. The other ammeter controller is the slave and does not generate switching control signals. One switching control signal is shared by 12 switches. The ammeter controller operates multiple ADCs synchronously and controls gains for the amplifiers.

### Main control module with constant voltage source

**Figure 4:**
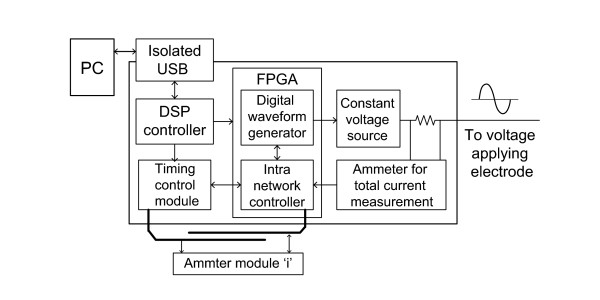
**Main control module.** Functional block diagram for the main control module including an isolated PC interface, constant voltage source, main controller, and intra-network controller. A timing control and network control signal are generated for each ammeter channel.

The main control module consists of a constant voltage source, a main controller implemented with a DSP (TMS320F2812, Texas Instruments, USA), an intra-network controller, and an isolated USB interface for communicating with a PC as shown in Figure 4. The intra-network controller and digital waveform generator are implemented on a FPGA. The intra-network controller arbitrates commands and data between the main controller and each of the ammeter controllers through multiple serial ports in a star configuration. The constant voltage source is implemented with a recent digital to analog converter (DAC) (AD9783, Analog Devices, USA) and operational amplifier. The DAC component includes two 16-bit DACs for generating sinusoid waveforms and two additional 10-bit DACs for compensating output offset and range controls. Analog outputs are summed and fed to a power amplifier (AD8018, Analog Devices, USA) capable of driving low distortion signals. We inserted a series current sensing resistor, 100*Ω*, at the voltage output. A voltmeter in the main control module measures the voltage difference across this series resistor to estimate the total amount of current injected into the tested object. A digital waveform generator controls the total amount of injected current below the safety level and records the magnitude and phase for compensating the measured data. The timing control module operates with the digital waveform generator to produce control signals for the switching module. The main controller manages the entire measurement process and communicates with a PC by a customized isolated USB connection consisting of a USB controller (C8051F320, Silicon Laboratory, USA) and digital signal isolator (ADuM4160, Analog Devices, USA).

### Calibration

The ground potential and virtual ground of the I-V converter input are not identical. The input resistances and capacitances from PCB routes, wires, connectors and switches also produce inconsistencies in different electrode connections. We remove such systematic artifacts due to non-ideal characteristics of the TAM system using following calibration method.

Calibration is performed using a rectangular saline tank where the top and bottom electrodes tightly fit the enclosure to prevent leakages. We calibrated TAM system with saline phantom in order to include the half cell potential and impedance characteristics of the electrodes. The calibration will not depend on the saline conductivity as long as the saline is within the measurable impedance range of the system. Inside the homogeneous saline tank, current density is uniform and parallel to the *z*-direction. First, we calculate phase compensation factors for each electrode to reduce the phase to zero, as expected for a purely resistive load. After applying phase compensation, we compute the averaged measurements of exit current from all current sensing electrodes in all possible switch combinations and find the magnitude scaling factors that make the exit current values equal to the averaged reference measurement value using fractional deviations. We store calibration factors and measured applied currents for each frequency because the amount of applied current is different depending on the operating frequency. This allows us to compensate the trans-admittance map with the measured amount of applied current and calibration factors at the specific operating frequency.

### Trans-admittance maps

The breast is situated between the two electrode plates as shown in Figure 1. To develop the trans-admittance map we consider the three-dimensional domain *Ω* of the breast bounded by its surface *∂Ω*. We apply a constant voltage of *A* volts with an angular frequency *ω* to the large electrode plate with contact area *Γ*_*S*_. The other electrode plate contains the 3600 current sensing electrodes at ground potential. These form a total contact area of *Γ*_*M*_. The electric field between the plates can be expressed as a complex voltage *V*(**r**) at position **r** = (*x*, *y*, *z*) in *Ω*. This satisfies the following mixed boundary value problem:

(1) $$\left\{\begin{array}{c} \nabla \cdot ((\sigma +\text{iωε})\nabla V(r))=0,\;r\in \Omega \\ V(r)=0,\;r\in \Gamma_{M}\\ V(r)=A,\;r\in \Gamma_{S}\\ \left(\sigma +\text{iωε})\nabla V(r)\cdot n(r)=0,\;r\in \partial \Omega \setminus (\Gamma_{S}\cup \Gamma_{M}\right) \end{array}\right)$$

where **n** is the unit outward normal vector to the boundary, *σ* = *σ*(**r**, *ω*) the conductivity and *ε* = *ε*(**r**,*ω*) the permittivity. Both *σ* and *ε* depend on **r** and *ω*. Denoting the real and imaginary parts of *V * by *v* = *ℜV* and *h* = *ℑV*, we have the following coupled system:

(2) $$\left\{\begin{array}{c} \nabla \cdot (\sigma \nabla v)-\nabla \cdot (\text{ωε}\nabla h)=0\;\;\mathrm{in}\;\Omega \\ \nabla \cdot (\text{ωε}\nabla v)+\nabla \cdot (\sigma \nabla h)=0\;\;\mathrm{in}\;\Omega \\ \;v=0\;\text{and}\;h=0\;\;\;\;\;\mathrm{on}\;\Gamma_{M}\\ \;v=A\;\text{and}\;h=0\;\;\;\;\mathrm{on}\;\Gamma_{S}\\ n\cdot \nabla v=0\;\text{and}\;n\cdot \nabla h=0\;\;\;\text{on}\partial \Omega \setminus (\Gamma_{S}\cup \Gamma_{M}). \end{array}\right)$$

In order to detect a lesion underneath the current sensing electrodes, we measure the distribution of exit currents through *Γ*_*M*_ as

(3) $$\begin{array}{cccc} -g(r):= & \underset{\text{real part}}{\underset{︸}{n\cdot (\sigma \nabla v-\text{ωε}\nabla h)}} & +i\;\underset{\text{imaginary part}}{\underset{︸}{n\cdot (\sigma \nabla h+\text{ωε}\nabla v)}} & ,\;\;r\in \Gamma_{M}. \end{array}$$

The distribution of exit currents *g* is called the trans-admittance map when an applying voltage is constant.

We suppose that there is a cancerous lesion *D* inside *Ω* as shown in Figure 1. The complex conductivity *τ*: = (*σ* + *iωε*) changes abruptly across the interface *∂D* and we assume

(4) $$\tau :=\sigma +\text{iωε}=\left\{\begin{array}{c} \sigma^{n}+\text{iω}\varepsilon^{n}\;\text{in the normal tissue}\;\Omega \setminus \bar{D}\\ \sigma^{c}+\text{iω}\varepsilon^{c}\;\text{in the cancerous lesion}\;D. \end{array}\right)$$

Both trans-admittance maps *g*_1_ and *g*_2_ are simultaneously acquired at two different frequencies *ω*_1_ and *ω*_2_, respectively, from the breast region Ω with a lesion *D*[22].

### Multi-frequency saline phantom experiment

**Figure 5:**
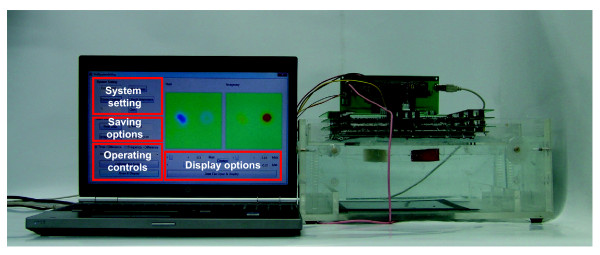
**The developed TAM system with user interface and saline phantom.** The developed TAM system with user interface software and saline phantom. The electrodes shown here are inverted with the large single voltage applying electrode plate on the bottom of the rectangular perspex tank. The array of current sensing electrodes are on the top surface of the tank to move them away from possible leaks or spillages.

The developed TAM system, shown in Figure 5, was evaluated in terms of noise, SNR, the waveform stability error of the constant voltage source, and the crosstalk between measurement channels. We first measured the noise levels of the ammeters by filling the gap between the top and bottom plates with 0.2 S/m saline. All measured deviations in exit currents from each electrode are considered noise. We measured the percentile stability error of the constant voltage source directly on the current sensing resistor, at multiple operating frequencies, and without using the phase-sensitive demodulator and I-V converter. Crosstalk was assessed between switches or ammeter channels. Finally, we measured the overall performance of the TAM system as SNR defined by the ratio of the mean value to the standard deviation of repeated measurements using the saline tank. We measured the real and imaginary components of exit currents to compute the SNR.

**Figure 6:**
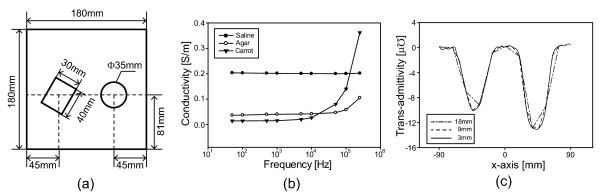
**Phantom setup and simulation results.(a)** Conductivity saline phantom with 2 anomalies (cuboid agar and cylindrical carrot), **(b)** conductivity spectrum for each testing material, **(c)** admittance of the cross-section over the center of the testing objects for various levels of electrode spacing. The total size of the phantom was constrained so an increased spacing corresponds to a simulation of less electrodes. i.e. the spacing of 3 *mm*, 9 *mm* and 18 *mm* correspond to 3600, 1200 and 600 electrodes.

In order to show the imaging performance of TAM system at multiple frequencies, we produce trans-admittance maps with objects which have different admittivity spectra and are similar to biological tissues. As the reference, we fill the 15*cm*deep phantom with 0.2 S/m saline. We inserted an agarose cuboid of 43 × 30 × 20 *mm*^3^ in the left side positioned at (-45 mm, -9 mm, -19 mm) while a cylindrical carrot with 35*mm* diameter and 20*mm* height is positioned on the right side (45 mm, -9 mm, -19 mm) as shown in Figure 6(a). The top surfaces of both objects are 9*mm* away from the current sensing electrode array. We obtain the current distribution data at 10 different frequencies, from 50 Hz to 500 kHz with a constant voltage of 1.4 V. Using a simple frequency difference algorithm we reconstruct frequency difference images from the measured trans-admittance maps obtained at different frequencies [25,26]. The admittance values from cross-sections of simulated maps of our phantom at different electrode spacing are shown in Figure 6(c). These demonstrate that there is improvement in the spatial resolution when decreasing the spacing of electrodes from 18 mm to 3 mm. In our test phantom geometry this corresponds to an increase from 600 to 3600 electrodes, thus providing motivation to increase the number of electrodes over previous TAM designs.

## Results

### Basic performance

**Figure 7:**
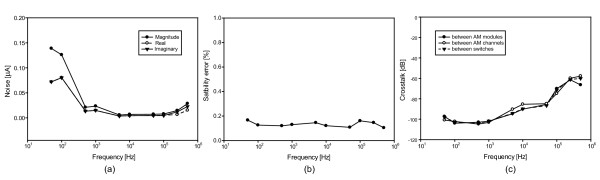
**Measurement of noise, amplitude stability and crosstalk.(a)** Noise spectra of magnitude, real, and imaginary components of measured exit currents, and **(b)** amplitude stability error of the constant voltage source. **(c)** Crosstalk between ammeter modules, adjacent ammeter channels, and neighboring switches in the switching module.

Figure 7(a) shows the noise amplitudes with saline between the two electrode plates at each operating frequency. The average magnitude of noise within the operating range was 38 nA. Noise was less than 5 nA for frequencies greater than 1 kHz. The amplitude stability error of the constant voltage source was 0.13±0.03% at all operating frequencies as shown in Figure 7(b). The crosstalk between neighboring channels on the switches, ammeter channels, and ammeter modules is shown in Figure 7(c). All operating frequencies produced SNR of better than 70 dB.

### Time-difference TAM images

**Figure 8:**
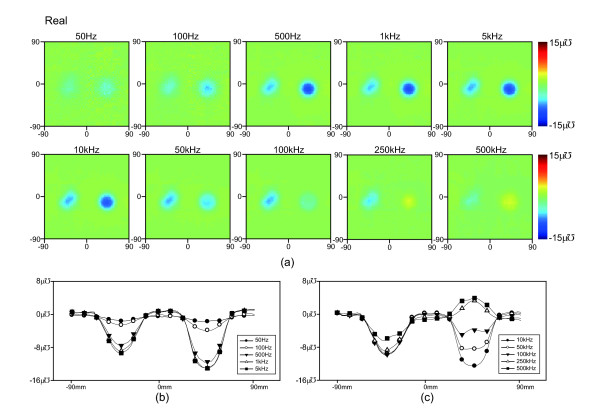
**Time difference images of the real component of admittance.(a)** Time difference real trans-admittance maps for the testing objects obtained at 10 different frequencies. The images are displayed for the full 180* mm* × 180 *mm* size of the electrode array using a RGB colorbar between −15 *uƱ* and + 15 *uƱ* as is customary in admittivity images. **(b)** and **(c)** Admittance of the cross section over the center of the testing objects at each frequency.

**Figure 9:**
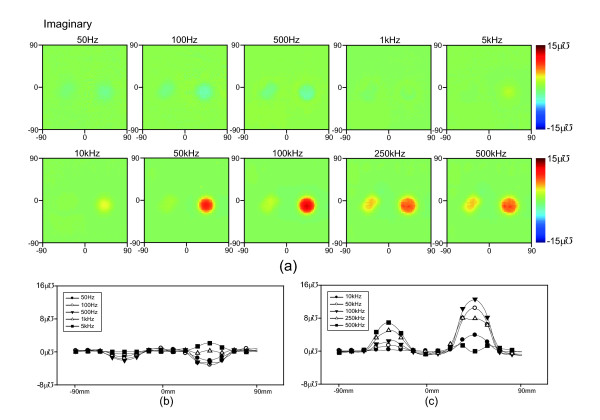
**Time difference images of the imaginary component of admittance.(a)** Time difference imaginary trans-admittance maps for the testing objects obtained at 10 different frequencies. The images are displayed for the full 180 *mm* × 180 *mm* size of the electrode array using a RGB colorbar between −15 *uƱ* and + 15 *uƱ* as is customary in admittivity images. **(b)** and **(c)** Admittance of the cross section over the center of the testing objects at each frequency.

Figures 8(a) and 9(a) show the time-difference real- and imaginary-part images at 10 different frequencies. Figures 8(b), 8(c), 9(b) and 9(c) show the central admittance cross section of the objects in each image. We measured reference conductivity of background saline, agar, and carrot at the operating frequencies of the TAM system using an impedance analyzer (1260A, AMETEK Inc., UK) as shown in Figure 7(b).

### Frequency-difference TAM images

**Figure 10:**
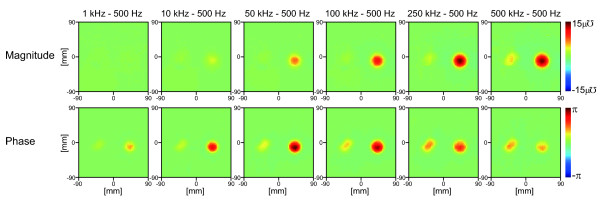
**Frequency difference images.(a)** Frequency difference images between 6 frequency pairs. The images are displayed for the full 180 *mm* × 180 *mm* size of the electrode array using a RGB colorbar between −15 *uƱ* and + 15 *uƱ* as is customary in admittivity images. Here we have displayed magnitude and phase images as they more clearly show the conductivity magnitude and phase change over frequency.

Figure 10 shows the magnitude and phase images of the trans-admittance distribution obtained at 6 different frequency pairs with respect to the 500 Hz.

## Discussion

### Basic performance

We developed a high density TAM system capable of producing trans-admittance projection images of conductive objects. We confirmed the performance of the TAM system with noise spectrum, amplitude stability of the constant voltage source, crosstalk on measurement channels, and SNRs within the operating frequency range of 50 Hz - 500 kHz. As shown in Figure 7 the system noise increased at the lower frequencies because the measurement time is related to the period of the operating frequency. The measurement time reduces with higher frequencies, example times are 64 s for 50 Hz, 1.6 s for 5 kHz and 12.8 ms for 500 kHz. These are not proportional due to our use of discrete clock division and the overhead of additional data transfers and synchronization wait times explained in a previous paper on an EIT system which forms the base architecture of the TAM system [25]. We used a filter to remove low frequency biosignals and DC components. The signal below 100 Hz is affected by this filter and drifts in conductivity and temperature. This was evident as we also saw the SNR of low frequencies degrading when the measurement time was increased.

The TAM system has a complex configuration with many current sensing electrodes and switches for measuring data with a small number of measurement channels. We examined crosstalk between neighboring channels on the switches, ammeter channels, and ammeter modules. Figure 7(c) shows that the average crosstalk was degraded from -98.5 dB below 10 kHz to -60 dB at 500 kHz. We examined crosstalk between switch input ports, adjacent ammeter channels, and ammeter channels belonging to different ammeter modules. They produced a similar frequency spectrum as shown in Figure 7(c). Most of the crosstalk was induced by the switching modules and little was added by the measurement routes on ammeter channels and boards.

We finally confirmed the total performance of TAM system as the SNR defined by the ratio of the mean value to the standard deviation of repeated measurements using the saline tank. As expected with a saline phantom, the real components of the measured data was dominant in the signal. All operating frequencies produced SNR of better than 70 dB.

### Time-difference TAM images

Figure 6(b) shows that the conductivity spectrum of agar had a smaller variation than carrot and that the conductivity of saline was relatively constant. The conductivity spectrum of carrot crossed over the conductivity spectrum of saline between 100 kHz and 250 kHz. The contrast of agar and carrot in the saline tank was degraded at frequencies greater than 100kHz, as expected from the real and imaginary spectra in Figures 8 and 9. Differences at frequencies less than 500 Hz may have been caused by the lower system performance at low frequencies as discussed previously. The contrast variation of the cylindrical carrot object showed a large difference between images and reversed above 250 kHz in close agreement with the reference spectra in Figure 6(b). Again at less than 500 Hz the contrast was affected by system performance. Interestingly for the carrot object, the imaginary images showed a larger difference than the real images. These results can also be observed in the cross sections of admittance plotted through the centre of the objects in Figures 8(b),(c) and 9(b),(c). The circular object creates a larger and flatter profile particularly at 100 kHz and 250 kHz while the square object creates a rounder profile. This change in profiles for different shapes at many frequencies may allow us to better identify the objects in an anomaly detection algorithm.

### Frequency-difference TAM images

We applied a simple frequency difference algorithm to the measurements from TAM system. Figure 10 shows the magnitude and phase images of the trans-admittance distribution obtained at 6 different frequency pairs with respect to the 500 Hz data. As expected the contrast of agar in the magnitude and phase images did not change significantly until the frequencies increased above 100 kHz, in agreement with the reference spectra in Figure 6(b). The contrast change of carrot in the magnitude and phase images over the frequency range also matched the reference spectra. These results show that the technique is feasible and gives us the confidence to move to tissue measurements.

In real human measurements, the spectrum will be affected by the skin-electrode interfaces since its characteristics are also varied by the operating frequency [27,28]. Although we have not tested this here we believe all impedance spectroscopy data will be deteriorated by this error, increasing the uncertainty of lesion detection inside the breast. An additional method to estimate the transfer function of the electrode interface is essential to remove this artefact.

Our system compares favourably with previous impedance imaging systems developed for breast cancer detection. In comparison with EIT systems, we found the significantly larger number of electrode channels enabled a resolution close to the electrode spacing of 3 mm while EIT systems with 72 electrodes or less have reported resolution on the centimeter scale [29,30]. Even with the larger number of channels, our system design allows a similar frame rate at 15 frames/s at 10 kHz and covers a larger frequency range than EIT systems reporting up to 2.6 frames/s and 1 kHz-1 MHz [30]. We were able to achieve these specifications without compromising crosstalk or noise which is reflected in our average SNR values of 70 dB (5-500 kHz) and 50 dB (50 Hz-1 kHz) which is worse than that achieved in EIT systems with significantly fewer electrodes (e.g. 100 dB (<100 kHz) and 60 dB (1 MHz) [29]). The main difference here may be due to the signal level differences where EIT systems measure voltages in the mV range, TAM requires the measurement of currents in the nA range.

This is reflected in a similar SNR to previous high channel count admittance based systems such as the T-scan which reported a SNR of 70 dB at 100 Hz and 40 dB at 100 kHz [31]. The T-scan system had a smaller frequency range and total probe size was much smaller at 72*mm* × 72*mm* with 256 electrodes. We, and others have shown in previous work that there is depth sensitivity in impedance measurements related to the separation between electrodes. In particular Seo *et al* (2004) [20], have shown that the depth information and transversal position can be estimated from the measured trans-admittance maps. This estimation is based on the amount of information and so is an additional motivation for increasing the number of electrodes. This is non-invasive and practical method to find anomaly inside the breast. This technique could be improved by repositioning the array to find more direct depth information. The dual plane sensors are similar to the mammography setup as we aim to compare mammography images with impedance images for the breast in the same shape and orientation. While the EIT methods where electrodes surround the entire breast may be more sensitive to depth information, this may be a disadvantage when comparing to the mammography configuration where the breast is situated between two plates. In addition EIT images have reduced resolution in the center of the image and away from the plane of the ring of electrodes [32]. The large array of closely spaced electrodes in our TAM array does not suffer from this variation in spatial resolution. The challenge we deal with in this paper is building the instrumentation and interface to such a large number of electrodes. We demonstrate that the imaging is successful and sensitive to different shaped objects in a saline tank.

## Conclusions

We found that the developed TAM system produced time-difference and frequency-difference trans-admittance images of both real and imaginary using carrot and agar objects in the saline phantom at 10 different frequencies. Contrast between background saline and anomalies changed according to the conductivity spectra of testing materials. We believe that the TAM system is capable of detecting a small anomaly in the breast from trans-admittance projection images with an average noise level of 38 nA and 70 dB SNR. The large number of current-sensing electrodes significantly improves the spatial resolution. We were not able to compare to the resolution of the conventional EIT method directly. However, the resolution of TAM with this large number of electrodes is visually superior to tomographic EIT.

A TAM system is expected to be a supplementary or alternative method to detect the breast cancer. The setup of the system is similar to the X-ray mammography with the breast placed between two plates. The plates are movable to accommodate breasts with different sizes and may be rotated to provide multiple images with different projection angles. In order to quantitatively analyze the ability to detect a small anomaly, we will formulate a mathematical framework to describe the relationship between the admittivity of the anomaly and measured spectroscopic trans-admittance maps.

## Abbreviations

TAM: Trans-admittance mammography; ADC: Analog to digital converter; EIT: Electrical impedance tomography; TAS: Trans-admittance scanner; FR4: Fire-retardant glass laminate susbtrate; PCB: Printed circuit board; I-V: Current to voltage converter; FPGA: Field programmable gate array; DAC: Digital to analog converter; USB: Universal serial bus.

## Competing interests

The authors declare that they have no competing interests.

## Authors’ contributions

MZ setup the system, performed and wrote up the experiments. HW developed the printed circuit boards and figures for the manuscript. AHMK wrote the system software and helped draft the manuscript. ALM assisted with analysis and gave critical revision for important intellectual content, EJW conceived the idea and revised the manuscript, and TIO designed the system and drafted the manuscript. All authors read and approved the final manuscript.

## References

[B1] Surowiec AJ Stuchly SS Barr JR Swarup A Dielectric properties of breast carcinoma and the surrounding tissues IEEE Trans Biomed Eng 1998 35 257 63 2834285 10.1109/10.1374

[B2] Jossinet J Schmitt M A review of parameters for the bioelectrical characterization of breast tissue Ann New York Acad of Sci 1999 873 30 41 10.1111/j.1749-6632.1999.tb09446.x 10372147

[B3] Silva JEMJP J J Classification of breast tissue by electrical impedance spectroscopy Med Biol Eng Comput 2000 38 26 30 10.1007/BF02344684 10829386

[B4] Hartov A Soni N Halter R Holder DS Breast cancer screening with electrical impedance tomography Electrical Impedance Tomography: Methods, History and Applications 2005 Bristol, UK: IOP Publishing 167 85

[B5] Holder DS Electrical Impedance Tomography: Methods, History and Applications 2005 Bristol, UK: IOP Publishing

[B6] Soleimani M Electrical impedance tomography system: an open access circuit design Biomed Eng OnLine 2006 5 28 10.1186/1475-925X-5-28 16672061 PMC1523342

[B7] Larson-Wiseman JL Early Breast Cancer Detection Utilizing Clustered Electrode Arrays in Impedance Imaging. PhD thesis 1998 Troy, NY, USA: RPI

[B8] Mueller JL Isaacson D Newell JC A reconstruction algorithm for electrical impedance tomography data collected on rectangular electrode arrays IEEE Trans Biomed Eng 1999 46 1379 86 10.1109/10.797998 10582423

[B9] Cherepenin V Karpov A Korjenevsky A Kornienko V Mazaletskaya A Mazourov D Meister J A 3D electrical impedance tomography (EIT) system for breast cancer detection Physiol Meas 2001 22 9 18 10.1088/0967-3334/22/1/302 11236894

[B10] Cherepenin V Karpov A Korjenevsky A Kornienko V Kultiasov Y Ochapkin M O T Meister J Three-dimensional EIT imaging of breast tissues: system design and clinical testing IEEE Trans Med Imag 2002 21 662 7 10.1109/TMI.2002.800602 12166863

[B11] Kerner TE Paulsen KD Hartov A Soho SK Poplack SP Electrical impedance spectroscopy of the breast: clinical imaging results in 26 subjects IEEE Trans Med Imag 2002 21 638 45 10.1109/TMI.2002.800606 12166860

[B12] Kao T Newell JC Saulinier GJ Isaacson D Distinguishability of inhomogeneities using planar electrode arrays and different patterns of applied excitation Physiol Meas 2003 24 403 11 10.1088/0967-3334/24/2/352 12812425

[B13] Kao T Isaacson D Newell JC Saulnier GJ A 3D reconstruction algorithm for EIT using a handheld probe for breast cancer detection Physiol Meas 2006 27 S1 11 10.1088/0967-3334/27/5/S01 16636401 PMC1513648

[B14] Kao T Saulnier GJ Xia H Tamma C Newell JC Isaacson D A compensated radiolucent electrode array for combined EIT and mammography Physiol Meas 2007 28 S291 9 10.1088/0967-3334/28/7/S22 17664644 PMC2423935

[B15] Choi MH Kao T Isaacson D Saulnier GJ Newell JC A reconstruction algorithm for breast cancer imaging with electrical impedance tomography in mammography geometry IEEE Trans Biomed Eng 2007 54 700 10 17405377 10.1109/TBME.2006.890139 PMC2759944

[B16] Boverman G Kao T Kulkarni R Kim BS Isaacson D Saulnier GJ Newell JC Robust linearized image reconstruction for multifrequency EIT of the breast IEEE Trans Med Imag 2008 27 1439 48 10.1109/TMI.2008.922187 PMC2568991 18815096

[B17] Kao T Boverman G Kim BS Isaacson D Saulnier GJ Newell JC Choi MH Moore RH Kopans DB Regional admittivity spectra with tomosynthesis images for breast cancer detection: preliminary patient study IEEE Trans Med Imag 2008 27 1762 8 10.1109/TMI.2008.926049 PMC2758037 19033092

[B18] Assenheimer M Laver-Moskovitz O Malonek D Manor D Nahliel U Nitzan R Saad A The T-Scan technology: electrical impedance as a disgnostic tool for breast cancer detection Physiol Meas 2001 22 1 8 10.1088/0967-3334/22/1/301 11236870

[B19] Scholz B Towards virtual electrical breast biopsy: space-frequency MUSIC for trans-admittance data IEEE Trans Med Imag 2002 21 588 95 10.1109/TMI.2002.800609 12166854

[B20] Seo JK Kwon O Ammari H Woo EJ Mathematical framework and anomaly estimation algorithm for breast cancer detection: electrical impedance technique using TS2000 configuration IEEE Trans Biomed Eng 2004 51 1898 906 10.1109/TBME.2004.834261 15536891

[B21] Ammari H Kwon O Seo JK Woo EJ T-Scan electrical impedance imaging system for anomaly detection SIAM J Appl Math 2004 65 252 66 10.1137/S003613990343375X

[B22] Oh TI Lee J Seo JK Kim SW Woo EJ Feasibility of breast cancer lesion detection using a multi-frequency trans-admittance scanner (TAS) with 10 Hz to 500 kHz bandwidth Physiol Meas 2007 28 S71 84 10.1088/0967-3334/28/7/S06 17664649

[B23] Kim S Lee J Seo JK Woo EJ Zribi H Multi-frequency trans-admittance scanner: mathematical framework and feasibility SIAM J Appl Math 2008 69 22 36 10.1137/070683593

[B24] Franco S Design with Operational Amplifiers and Analog Integrated Circuits 2002 3rd. ed. NY, USA: McGraw-Hill

[B25] Oh TI Koo H Lee KH Kim SM Lee J Kim SW Seo JK Woo EJ Validation of a multi-frequency electrical impedance tomography (mfEIT) system KHU Mark1: impedance spectroscopy and time-difference imaging Physiol Meas 2008 29 295 307 10.1088/0967-3334/29/3/002 18367806

[B26] Jun SC Kuen J Lee J Woo EJ Holder D Seo JK Frequency-difference EIT (fdEIT) using weighted difference and equivalent homogeneous admittivity: validation by simulation and tank experiment Physiol Meas 2009 30 10 1087 [ http://stacks.iop.org/0967-3334/30/i=10/a=009] 10.1088/0967-3334/30/10/009 19738319

[B27] Oh TI Yoon S Kim TE Wi H Kim KJ Woo EJ Sadleir RJ Nanofiber web textile dry electrodes for long-term biopotential recording IEEE T Biomed Circ Sin press 10.1109/TBCAS.2012.2201154 23853303

[B28] Woo E Hua P Webster J Skin impedance measurements Med Biol Eng Comput 1992 30 97 102 10.1007/BF02446200 1640763

[B29] Osterman KS Kerner TE Williams DB Hartov A Poplack SP Paulsen KD Multifrequency electrical impedance imaging: preliminary in vivo experience in breast Physiol Meas 2000 21 99 10.1088/0967-3334/21/1/313 10720005

[B30] Saulnier G Liu N Tamma C Xia H Kao TJ Newell J Isaacson D IEEE Washington DC An Electrical Impedance Spectroscopy System for Breast Cancer Detection Engineering in Medicine and Biology Society, 2007. EMBS 2007. 29th Annual International Conference of the IEEE 2007 2007 4154 4157 10.1109/IEMBS.2007.4353251 18002917

[B31] Assenheimer M Laver-Moskovitz O Malonek D Manor D Nahaliel U Nitzan R Saad A The T-SCAN TM technology: electrical impedance as a diagnostic tool for breast cancer detection Physiol Meas 2001 22 1 10.1088/0967-3334/22/1/301 11236870

[B32] Metherall P Barber DC Smallwood RH Brown BH Three-dimensional electrical impedance tomography Nature 1996 380 6574 509 512 10.1038/380509a0 8606768

